# Expression of cytoplasmic and nuclear Survivin in primary and secondary human glioblastoma

**DOI:** 10.1038/sj.bjc.6602904

**Published:** 2005-12-13

**Authors:** D Xie, Y X Zeng, H J Wang, J M Wen, Y Tao, J S T Sham, X Y Guan

**Affiliations:** 1 State Key Laboratory of Oncology in Southern China, Cancer Center, Sun Yat-Sen University, Guangzhou, China; 2 Department of Neurosurgery, the First Affiliated Hospital, Sun Yat-Sen University, Guangzhou, China; 3 Department of Pathology, Zhong Shan Medical College, Sun Yat-Sen University, Guangzhou, China; 4 Department of Clinical Oncology, University of Hong Kong, Hong Kong, China

**Keywords:** glioblastoma multiform, Survivin, immunohistochemistry, apoptosis

## Abstract

Clinically, human glioblastoma (GBM) may develop *de novo* or from a low-grade glioma (secondary GBM), and molecular alterations in the two pathways may differ. This study examined the status of Survivin expression and apoptosis in 30 primary and 26 secondary GBMs. Our results show that cytoplasmic Survivin positivity was significantly (*P*<0.001) more frequent in primary GBMs (83%) than that in secondary GBMs (46%). In addition, an inverse correlation of cytoplasmc Survivin positivity with GBM apoptotic index, and a positive association between cytoplasmic Survivin and size of the tumours were observed. These results suggest that cytoplasmic Survivin, via its antiapoptotic function, may be involved in the tumorigenesis of many primary GBMs, but only in a small fraction of secondary GBMs. Furthermore, the overall progression times from low-grade precursor lesions to secondary GBMs were significantly shorter (*P*<0.05) in cytoplasmic Survivin-positive cases (mean, 15.6 months) than those in Survivin-negative cases (mean, 23.8 moths), and the positive expression level of Survivin in cytoplasm was upregulated in most secondary GBMs when compared to matched pre-existing low-graded lesions. These results suggest that the increased accumulation of Survivin in the cytoplasm of more malignant glioma cells may prove to be a selective advantage, thus accelerating progression to a more aggressive phenotype.

Survivin is located on chromosome 17q25. It was initially identified as a gene with structural homology to a family of genes known as inhibitors of apoptosis (IAPs) (Ambrosini *et al*, 1997). Survivin is known to be normally expressed at high levels in fetal tissue but absent in most normal adult differentiated cells (Adida *et al*, 1998). It is expressed extensively in common human cancers, such as colorectal carcinoma (Kawasaki *et al*, 1998), lung cancer (Monzo *et al*, 1999), and oesophageal carcinoma (Kato *et al*, 2001), and the increased expression of Survivin appears to be associated with the aggressive nature and/or poor prognosis of the above cancers. It is thought that Survivin enhances survival of tumour cells primarily through its antiapoptotic function via direct blocking of the terminal effecter cell-death proteases, caspases-3 and -7, which are distinct from the BCL-2 family of antiapoptotic proteins (Shin *et al*, 2001). In human gliomas, some reports have documented that the increased expression of cytoplasmic Survivin, detected by immunohistochemistry (IHC), in gliomas was correlated with an ascending pathological grade of tumour (Sasaki *et al*, 2002; Jiao *et al*, 2004). In addition, in glioblastoma multiform (GBM, WHO, grade IV), patients with detectable Survivin expression by Western blot analysis have been observed to have significantly shorter overall survival times compared with those without detectable expression (Chakravarti *et al*, 2002). These results suggest that Survivin might be involved in tumorigenesis and progression of GBM.

It has been suggested that the development of GBM could be along two distinct pathogenic pathways (Watanabe *et al*, 1996). Primary GBM may arise from *de novo* after a short clinical history without an identifiable less-malignant precursor lesion and frequently contains EGFR amplification/overexpression, PTEN mutation, homozygous p16 deletion, and loss of heterozygosity (LOH) on chromosome 19q. Secondary GBM often develops more slowly from a low-grade or anaplastic astrocytoma. *P53* mutations and LOH on chromosome 10q are often observed, while amplification/overexpression of EGFR is most often lacking (Watanabe *et al*, 1996; Nakamura *et al*, 2000). This suggests that primary and secondary GBMs develop differently on a genetic level. However, whether or not the expression of Survivin is different between primary and secondary GBMs and the potential role of Survivin in tumorigenesis of GBMs in different pathogenic pathways have not been described to date.

In the present study, we evaluated the protein expression of Survivin by IHC in a total of 56 GBMs, including 30 primary and 26 secondary GBMs, and in 15 matched low-grade precursor lesions (grade II or III gliomas) of secondary GBMs. Since Survivin has both nuclear and cytoplasmic targets, which appear to serve different functions (Mahotka *et al*, 2002), two different commercially available Survivin antibodies, which recognise predominantly nuclear and cytoplasmic forms of Survivin, were used (Altura *et al*, 2003). Nuclear Survivin has been suggested to play an important role in chromosomal segregation during mitosis (Uren *et al*, 2000; Honda *et al*, 2003), while cytoplasmic Survivin has been characterised as antiapoptotic (O'Connor *et al*, 2002). In addition, the TUNEL assay was used to detect the apoptotic status of the GBMs and apoptotic status was correlated with the expression of different Survivin forms. Our results, which extend previous results of others (Chakravarti *et al*, 2002; Sasaki *et al*, 2002; Jiao *et al*, 2004) that examined Survivin expression in human gliomas, are intended to shed light on our understanding of the developmental genetic mechanisms of primary and secondary GBMs.

## MATERIALS AND METHODS

### Patients and tissues specimens

In this study, 56 cases of GBM patients, whose clinical and pathological information were available in detail, were selected from the First Affiliated Hospital of Sun Yat-Sen University (SYSU, Guang Zhou, China) from January 1990 to January 2000. The age of patients at the time of surgery ranged from 9 to 67 years, and the male/female ratio was 2.1 : 1. In all, 45 tumours were localised in the hemispheres, six tumours were in the basal ganglia, and five tumours were distributed in the brain stem. A total of 35 GBMs were larger than 5 cm in diameter, while 21 GBMs were no more than 5 cm in diameter.

Of the 56 patients studied, 30 were primary GBMs, 26 were secondary GBMs. Diagnostic criteria for primary and secondary GBM were according to a previous report (Watanabe *et al*, 1996). Primary GBM was defined on the basis of clinical history of less than 3 months and patient's pathological diagnosis at the first onset of GBM. The diagnosis of secondary GBM required at least two biopsies and clinical as well as histological evidence of progression from low-graded (II–III) glioma, but it was pathologically diagnosed as GBM at the last operation. The clinical history of secondary GBM was at minimum longer than 3 months. The biopsy specimens, processed into paraffin blocks, of both primary and secondary GBMs were obtained from the Department of Pathology and Neurological Surgery, the First Affiliated Hospital of SYSU. In addition, in these GBM series, 15 matched precursor lesions (low-graded glioma) for secondary GBMs were also available, in which 10 cases were grade II glioma and five were anaplastic glioma (grade III).

### Immunohistochemistry

Immunohistochemistry studies were performed using the SABC method as previously described (Xie *et al*, 2003) with slight modifications. For antigen retrieval, slides were microwave-treated in a 50 mM citrate buffer and boiled (pH 6.0) for 10 min. Nonspecific binding was blocked with 10% normal rabbit serum for 20 min. The tumour slides were incubated at 4°C overnight in a moist chamber with one of the two polyclonal anti-Survivin antibodies (NB-500-201 K3, Novus Biologicals, Littleton, CO, USA, 1 : 300 dilution and SC-10811, Santa Cruz Biotechnology, Santa Cruz, CA, USA, 1 : 400 dilution). Each antibody was known to detect predominantly cytoplasmic (NB-500-201 K3) or the nuclear (SC-10811) forms of Survivin (Altura *et al*, 2003). Negative controls were established by replacing the primary antibody with a normal rabbit Ig.

### Evaluation of IHC results

Each IHC slide was assessed in 10 high-powered fields at × 40 magnification, and Survivin staining was evaluated semiquantitatively. A staining score (to ++++) was obtained as the proportion of immunopositive cells in each tumour. Expression of Survivin was scored as ‘−’ for negative expression, ‘+’ for positive staining in <25 cells, ‘++’ for positive staining in 25–50% cells, ‘+++’ for positive staining in 51–75% cells, and ‘++++’ for positive staining in >75% cells.

### TUNEL assay

**Figure 1:**
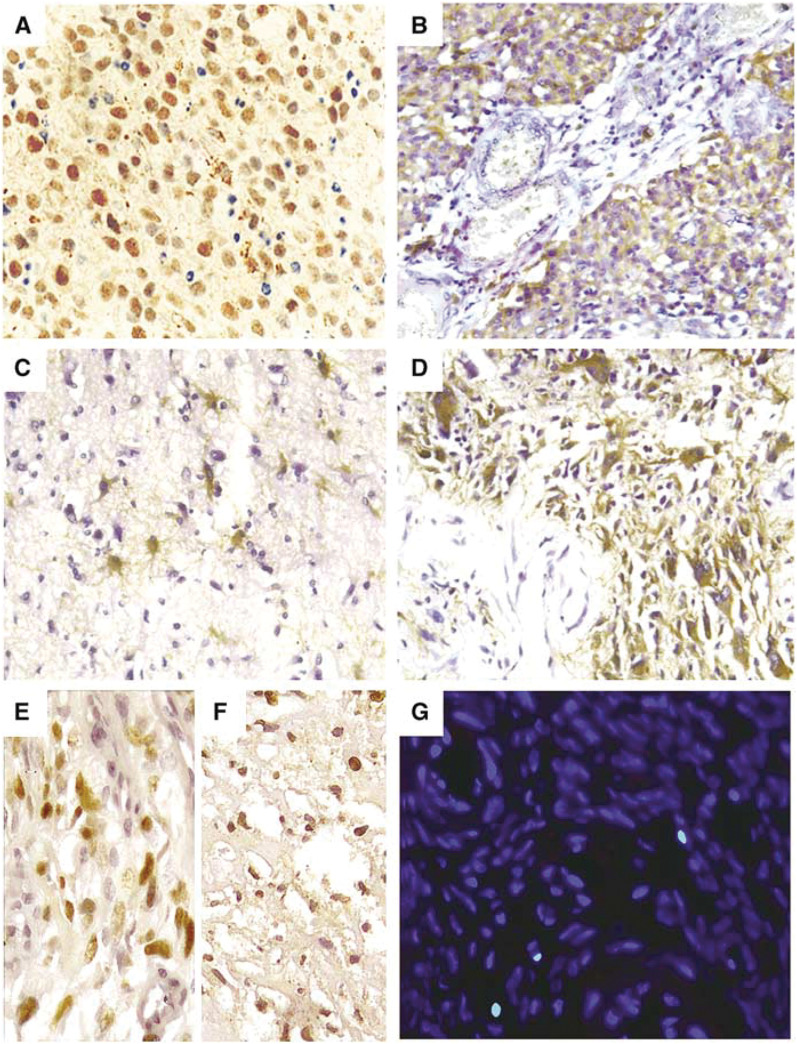
Immunohistochemical staining of Survivin and fluorescent TUNEL staining in human gliomas (**A–G**). A primary glioblastoma showed a high level (++++) expression of nuclear Survivin (**A**). High staining score (++++) of cytoplasmic Survivin was detected in another primary glioblastoma (**B**). Moderate expression level (++) of cytoplasmic Survivin was detected in a pre-existing low-graded (grade II) glioma (**C**), which was obviously lower than that in its paired secondary glioblastoma with a staining score of ++++ (**D**). High-level (++++) expression of nuclear Survivin was detected in a secondary glioblastoma (**E**) and its matched pre-existing grade II glioma (**F**). Three apoptotic cells with clear nuclear staining (green color) were examined in a primary glioblastoma (**G**).

The fluorescent TUNEL staining was performed using a Death Detection kit (Roche Diagnostic GmbH, Mannheim, Germany) according to the manufacturer's instructions. Briefly, the rehydrated section was microwave-treated in 10 mM citrate buffer (pH 6.0) for 5 min. After washing in PBS, the specimen was incubated with a mixture of TdT solution (enzyme solution) and FITC-lablled dUTP solution (fluorescence labled solution) in a humidified chamber in the dark at 37°C for 60 min. After washing, the slide was examined with a Zeiss Axiophot fluorescence microscope. Negative controls were obtained by replacing the TdT solution with distilled water. The presence of clear nuclear staining (TUNEL positive, green colour) was indicative of apoptotic cells (Figure 1G). At least 1000 tumour cell nuclei were examined. The number of TUNEL-positive tumour cell nuclei was counted and the apoptotic index (AI) was determined as the percentage of apoptotic cells in the tumour. For evaluation of the TUNEL staining, the mean value of AI of all samples under study was often used as a cutoff value (Yamasaki *et al*, 1997). In this study, the mean value of AI for all GBM samples was 0.76, thus, tumours were classified into two groups according to their AI: low AI group (AI<0.76) and high AI group (AI⩾0.76).

### Statistical analysis

The differences in Survivin expression between primary and secondary GBMs and the correlation of Survivin in GBM with patient's clinicopathological parameters and cell apoptosis were assessed by *χ*^2^ test. Unpaired *t*-test was used to assess the statistical significance of the differential recurrent times from precursor lesion to secondary GBM, between groups with and without Survivin expression. *P*-values of <0.05 were considered to be significant.

## RESULTS

### Survivin expression in primary and secondary GBMs

Using two different Survivin antibodies, we were able to detect predominantly cytoplasmic (NB-500–201 K3, Figure 1B–D) and nuclear (SC-10811, Figure 1A, E, and F) forms of Survivin in GBMs by IHC, although both antibodies had some overlapping reactivity. The results of Survivin expression in GBMs are outlined in Tables 1 and 2. Of the 56 GBMs, 37 (66%) had cytoplasmic positive staining of Survivin and 43 (77%) had nuclear positive staining of Survivin. For the expression of Survivin in primary and secondary GBMs, the frequency of cytoplasmic positive staining of Survivin detected in primary GBMs was 83% (25 out of 30), which was significantly greater than that in secondary GBMs (46%, 12 out of 26) (*P*<0.001). In contrast, nuclear expression of Survivin showed no significant difference between primary and secondary GBMs (*P*=0.51), that is, the rate of nuclear positive staining of Survivin in the primary GBMs was 73% (22 out of 30), and in the secondary GBM group, 81% (21 out of 26).

### Survivin expression in secondary GBMs and matched precursor lesions

Owing to the suggestion that increased expression of Survivin in human gliomas is positively correlated with an ascending pathological grade of tumour (Sasaki *et al*, 2002), we further evaluated the expression pattern of Survivin between 15 pairs of secondary GBMs and their pre-existing lower grade lesions. Overall, the expression level (staining score) of nuclear and cytoplasmic Survivin between the 15 matched secondary GBMs and precursor lesions was concordant. That is, of the seven secondary GBMs showing cytoplasmic positive of Survivin, six paired precursor lesions also had positive cytoplasmic Survivin staining and of the 11 secondary GBMs showing nuclear Survivin positive staining, each of the paired precursor lesions also showed positive nuclear Survivin (Table 3). The expression level (staining score) of cytoplasmic Survivin in the secondary GBMs was higher than that in paired pre-existing lower grade lesions in five of the seven paired cases (Table 3, Figure 1C and D). A difference in the expression level of nuclear Survivin between secondary GBMs and paired pre-existing lower grade lesions was not observed (Figure 1E and F).

### Association of Survivin expression with clinicopathological features

A potential association between GBM Survivin expression and several known clinicopathological features, including patient's age, gender, tumour site, and tumour size, was further examined. No significant association was observed between Survivin nuclear expression and any of the patient's clinicopathological parameters (*P*>0.05). In contrast, a significant association of Survivin cytoplasmic expression with tumour size was observed (*P*<0.01). Thus, 80% (28 out of 35) of the large-sized GBMs (tumour size ⩾5 cm in diameter) were found to have positive expression of cytoplasmic Survivin, while only 43% (nine out of 21) of the small-sized GBMs (<5 cm in diameter) had positive expression of cytoplasmic Survivin. Cytoplasmic Survivin expression did not show a significant association with any other clinicopathological parameters of the patient (*P*>0.05). In secondary GBMs, the mean progression times in months from precursor lesion onset to secondary GBMs was significantly shorter in cytoplasmic Survivin-positive cases (mean, 15.6 months) than that in cytoplasmic Survivin-negative cases (mean, 23.8 moths, *P*<0.05), (Table 2).

### Correlation of Survivin expression with cell apoptosis

In this study, the TUNEL assay was used to study the status of apoptosis in all 56 GBMs. High AI (AI⩾0.76) was detected in 23 out of 56 (41%) of the tumours. Furthormore, a significant inverse correlation between Survivin cytoplasmic expression and tumour apoptosis was observed (*P* <0.001), that is, in the GBMs with positive expression of cytoplasmic Survivin, 84% (31 out of 37) were observed to have a low AI (AI<0.76), whereas in the GBMs showing negativity of cytoplasmic Survivin (17 out of 19, 89%), a high AI (AI⩾0.76) was found (see Table 1 and 2). No significant correlation was observed between nuclear Survivin expression and GBM AI.

## DISCUSSION

GBM, the most malignant of human brain glial tumours, may develop *de novo* (primary GBM) or via another pathogenic pathway, that is, from a low-grade glioma (secondary GBM) and the molecular alterations leading to the development of GBMs may differ (Nakamura *et al*, 2000). A previous report has documented a high frequent positive expression of Survivin in primary GBMs (Das *et al*, 2002). However, the expression pattern of this protein in secondary GBMs has not been described. In this study, the differences of Survivin expression between primary and secondary GBMs were examined by IHC using two different antibodies, one which detects predominantly cytoplasmic Survivin and one which detects predominantly nuclear forms of Survivin. Our results provide evidence that the expression pattern of nuclear Survivin was similar between primary and secondary GBMs, but, in contrast, cytoplasmic Survivin positivity was significantly more frequent in primary GBMs than that in secondary GBMs. It appears, therefore, that cytoplasmic expression of Survivin may provide additional insight into the developmental genetic mechanisms of GBM in different pathogenic pathways.

As discussed in detail in several intracellular localisation studies of Survivin protein, at present, at least three different splice variants of Survivin (ie, Survivin, Survivin delta Ex 3, and Survivin-2B) have been identified and have different subcellular localisations. Survivin and Survivin 2B isoforms localise predominantly to the cytoplasm, whereas the delta Ex 3 isoform is preferentially localised in the nucleus (Mahotka *et al*, 2002). Nuclear and cytoplasmic pools of Survivin have distinct roles. Cytoplasmic Survivin has been characterised as antiapoptotic (O'Connor *et al*, 2002), while nuclear Survivin has been proposed to serve in the maintenance of the integrity of the mitotic spindle (Uren *et al*, 2000). Germane to previous studies (Jiao *et al*, 2004), we found an inverse correlation between the cytoplasmic expression of Survivin and cell apoptosis in our GBM cohorts, thus suggesting an antiapoptotic function of the cytoplasmic form of Survivin in GBMs. In addition, we observed a significant association between Survivin cytoplasmic expression and GBM tumour size. The positive of cytoplasmic Survivin expression was found more frequently in large-sized GBMs (tumour size ⩾5 cm in diameter) than that in small-sized GBMs, the latter in which high-level cell apoptosis was more common. These results suggest that the antiapoptotic activity of cytoplasmic Survivin may be responsible, at least in part, for the growth and aggressive behaviour of GBM tumour cells.

In secondary GBMs, we found that the positive expression level of cytoplasmic Survivin was increased in most of secondary GBMs when compared to their matched low-grade tumours from the same patient, although the frequency of cytoplasmic Survivin positivity was similar to that in paired pre-existing low-graded lesions. In addition, the overall time in months of low-grade precursor lesion progression to secondary GBM was substantially less in cytoplasmic Survivin-positive cases than that of Survivin-negative cases. These results suggest a potential tumorigenic role of an upregulated expression of cytoplasmic Survivin along the linear progression model of low-grade lesions to secondary GBM. This also implies the possibility that the increased accumulation of Survivin in cytoplasm opposes apoptotic death in the early less-malignant glioma cells and thus may provide a selective advantage and acceleration of progression from a lower grade glioma into a higher grade more malignant phenotype. These observations, in addition, may shed light on explaining why many gliomas, even at their earliest stages, are so resistant to conventional apoptosis-related chemotherapy and radiation. However, it also appears that cytoplasmic Survivin accumulation is probably just one of several mechanisms that glioma cells may use to evade apoptosis, as we observed that more than 50% of secondary GBMs and their pre-existing low-grade gliomas failed to express cytoplasmic Survivin.

An intriguing question that evolved in our studies is why is the rate of cytoplasmic Survivin in secondary GBMs significantly lower than that in primary GBMs? Although the pathogenic pathways leading to the development of GBMs may differ, almost all GBMs tend to possess active proliferation, high invasiveness and angiogenesis potential, resistance to conventional cytotoxic treatments and most of these patients have survival times less than 1 year (Davis *et al*, 1999). These similar neuropathologic and behavioural features of both primary and secondary GBMs imply that the criteria of clinicopathological classification for GBM may not reflect their malignant potential. In addition, it is also true that some of the primary GBMs may have rapidly progressed from lower grade gliomas, which often are clinically asymptomatic. Indeed, the positive expression levels of cytoplasmic Survivin in our primary GBMs were similar to that in our secondary GBMs, although the frequency of positivity was substantially higher in the former than in the later. These observations, collectively, prompt us to hypothesise that the high frequency of cytoplasmic Survivin in primary GBM may increase the barriers to cell apoptosis, accelerate the oncogenic process of primary GBM, in particular, facilitate the development of primary GBM in a short period without an identifiable less-malignant pre-existing lesion. Thus, if the above hypothesis has validity, it is not so difficult to understand why Survivin accumulation in cytoplasm is much more frequent in primary GBMs than that in secondary GBMs.

For the nuclear form of Survivin, we found that its expression pattern in secondary GBMs and paired low-graded precursor lesions was almost concordant. This was consistent, in part, with other observations, where no differential expression of nuclear Survivin was observed between independent low-graded gliomas and GBMs (Kleinschmidt-DeMasters *et al*, 2003). This suggests the possibility that the positive expression of the nuclear form of Survivin may be an early molecular event involved in the development of secondary GBMs. Although the potential role of nuclear Survivin and its mechanism of action in human cancers are still unclear, Honda *et al* (2003) recently have observed that in the nucleus, Survivin interacted with INCENP and aurora B kinase; these enzymes have an important role in chromosomal segregation during mitosis. Moreover, knockout or inhibition of Survivin has been shown to result in multinucleated and polyploid cells, which is a characteristic of mitotic arrest (Speliotes *et al*, 2000). In our GBM studies, the majority of GBMs showed an aneuploid DNA content (Xie *et al*, 2005), and, furthermore, a close association between nuclear Survivin positivity and tumour aneuploidy was observed (data not shown). These results suggest that the nuclear form of Survivin in GBMs may influence mitotic events and subsequently facilitate chromosomal instability. It is known that genetic instability can cause cytogenetic heterogeneity within a number of tumour types, including gliomas (Harada *et al*, 1998). Thus, it is quite possible that the widespread chromosomal instability associated with nuclear expression of Survivin observed in many GBMs can determine certain GBM histopathological characteristics, such as the presence of tumour cell heterogeneity and/or multiform tumour cells. However, the prognostic significance of nuclear Survivin among human cancers does vary in different tumour types. Recently, nuclear Survivin positivity has been reported to be predictive of poor survival in patients with oesophageal carcinoma and non-small-cell lung cancer (Grabowski *et al*, 2003; Lu *et al*, 2004). In contrast, high nuclear Survivin expression has been shown to be an independent indicator of a favourable prognosis in osteosarcoma, breast cancer, and gastric carcinomas (Okada *et al*, 2001; Kennedy *et al*, 2003; Trieb *et al*, 2003) and, moreover, it has been associated with a less-progressive cytologic grade in pediatric ependymomas and choroid plexus tumours of the brain (Altura *et al*, 2003). As regards to human glioma, more recently, the index of nuclear expression of Survivin has been observed to have a strong reverse association with the overall survival time of glioma patients in different grades (Uematsu *et al*, 2005). However, no prognostic impact of nuclear expression of Survivin in GBM was observed (Preusser *et al*, 2005). These observations suggest that the action of nuclear Survivin in tumour cells may be tumour specific. Further studies are clearly needed to elucidate the underlying function of nuclear Survivin in GBMs as well as in other human cancers.

In summary, in the present study, we describe for the first time the expression pattern of both nuclear and cytoplamic forms of Survivin in primary and secondary GBMs. Our results indicate that these different forms of Survivin (nuclear and cytoplasmic) have different functions in influencing the malignant behaviour of human glioma cells and these would have a profound effect on the development and/or progression of primary and secondary GBMs. Further studies designed to determine whether or not there is an association between cytoplasmic and/or nuclear expression of Survivin in GBM and GBM patients' clinical outcomes are clearly in order.

## Figures and Tables

**Table 1:** Status of Survivin expression and apoptotic index in primary GBMs

**Case no.**	**Age/sex**	**Tumour size (cm)**	**Cytoplasmic Survivin^a^**	**Nuclear Survivin^a^**	**AI^b^**
1	11/M	5.7	+++	−	Low
2	15/M	3.1	−	++	High
3	16/F	6.3	++++	+++	Low
4	16/M	7.8	+++	+++	Low
5	19/M	4.3	++	+	Low
6	23/M	5.6	++++	++++	Low
7	27/F	7.3	++++	+++	High
8	28/M	8.5	++++	++++	Low
9	30/F	6.4	−	−	High
10	33/M	5.1	+++	++	High
11	35/M	3.5	++++	−	Low
12	35/F	5.2	−	++++	High
13	36/M	7.5	++++	++++	Low
14	37/M	10.2	+++	++	High
15	38/M	5.8	+++	−	Low
16	39/M	7.6	++++	+++	High
17	39/M	5.5	+	+++	Low
18	40/M	5.9	+++	−	Low
19	40/M	2.7	−	+	High
20	41/M	5.8	+	−	Low
21	41/M	3.4	+++	++++	Low
22	42/F	5.8	++++	++++	Low
23	43/M	4.1	++++	+++	Low
24	45/M	11.3	+++	+++	Low
25	46/M	4.4	++++	++	High
26	49/M	9.2	++	++++	Low
27	52/F	4.8	−	+	High
28	53/M	5	++	++++	Low
29	61/M	6.8	++++	−	Low
30	67/F	12	+++	−	Low

a Expression of Survivin detected by immunohistochemistry were scored as ‘−’ for negative expression, ‘+’ for positivity in <25 cells, ‘++’ for positivity in 25–50% cells, ‘+++’ for positivity in 51–75% cells, and ‘++++’ for positivity in >75% cells.

b AI=apoptotic index; AI⩾0.76 were scored as ‘high’, AI<0.76 were scored as ‘low’.

**Table 2:** Status of Survivin expression and apoptotic index in secondary GBMs

						**Preexisting lesion**	
**Case no.**	**Age/sex**	**Tumour size (cm)**	**Cytoplasmic Survivin^a^**	**Nuclear Survivin^a^**	**AI^b^**	**Grade**	**Progressing time^c^**
1	9/M	6.3	−	+++	Low	II	18
2	15/M	2.2	−	−	High	II	36
3	16/M	5.8	++++	+++	Low	II	31
4	17/F	3.7	++++	++	High	III	11
5	17/M	8.8	−	+++	High	II	33
6	18/F	6.5	+++	++++	Low	III	4
7	23/F	4.9	−	++++	High	II	48
8	25/M	6.4	−	−	High	II	23
9	27/M	3.1	−	+	High	III	18
10	29/M	10.2	+++	−	Low	II	31
11	33/F	2.9	−	+	High	III	27
12	35/M	7.2	++++	+++	Low	II	22
13	38/F	6.3	++	+++	Low	II	21
14	39/M	4.3	−	++	High	III	32
15	41/M	9.3	++++	−	Low	III	7
16	43/F	5.8	−	++	High	II	24
17	43/M	3.0	−	++	High	II	11
18	44/F	5.1	++++	+++	Low	III	18
19	43/M	4.9	−	−	Low	II	26
20	45/F	11.3	−	++	High	II	8
21	45/M	7.7	+++	+++	Low	II	9
22	47/M	6.1	++++	+++	Low	III	6
23	49/F	2.3	−	+	High	II	24
24	55/F	9.7	++++	++++	Low	III	16
25	60/M	3.8	−	++++	High	II	5
26	61/M	4.7	++++	++	Low	III	11

a Expression of Survivin detected by immunohistochemistry were scored as ‘−’ for negative expression, ‘+’ for positivity in <25 cells, ‘++’ for positivity in 25–50% cells, ‘+++’ for positivity in 51–75% cells, and ‘++++’ for positivity in >75% cells.

b AI=apoptotic index; AI⩾0.76 were scored as ‘high’, AI<0.76 were scored as ‘low’.

c The times (months) form the first onset of low-grade glioma progressing to secondary GBM.

**Table 3:** The expression of cytoplasmic and nuclear Survivin in 15 paired secondary GBMs and pre-existing lesions^a^

	**Cytoplasmic Survivin**		**Nuclear Survivin**	
**Case no.**	**Secondary GBM**	**Pre-existing lesion**	**Secondary GBM**	**Pre-existing lesion**
1	−	−	+++	+++
3	++++	++	+++	++++
4	++++	+	++	++
5	−	−	+++	+++
7	−	−	++++	++++
8	−	−	−	−
10	+++	−	−	−
11	−	−	+	++
15	++++	+++	−	−
17	−	−	++	++
19	−	−	−	−
21	+++	+++	+++	+++
22	++++	++	+++	++
25	−	−	++++	++++
26	−	−	++	++

a Expression of Survivin detected by immunohistochemistry were scored as ‘−’ for negative expression, ‘+’ for positivity in <25 cells, ‘++’ for positivity in 25–50% cells, ‘+++’ for positivity in 51–75% cells, and ‘++++’ for positivity in >75% cells.
